# Suppression of the T-dependent antibody response following oral exposure to selected polycyclic aromatic compounds in B6C3F1/N mice

**DOI:** 10.3389/ftox.2025.1558639

**Published:** 2025-03-06

**Authors:** Victor J. Johnson, Cynthia V. Rider, Michael I. Luster, Cynthia J. Willson, Shawn Harris, Billie Stiffler, James Blake, Esra Mutlu, Veronica Godfrey, Brian Burback, Reshan Fernando, Suramya Waidyanatha, Gary R. Burleson, Dori R. Germolec

**Affiliations:** ^1^ Burleson Research Technologies, Inc., Morrisville, NC, United States; ^2^ Division of Translational Toxicology, National Institute of Environmental Health Sciences, Research Triangle Park, NC, United States; ^3^ Integrated Laboratory Systems, LLC, An Inotiv Company, Morrisville, NC, United States; ^4^ DLH, LLC, Bethesda, MD, United States; ^5^ Battelle, Columbus, OH, United States; ^6^ RTI International, Research Triangle Park, NC, United States

**Keywords:** immunotoxicity, immunosuppression, polycyclic aromatic hydrocarbons (PAH), benzo(a)pyrene, phenanthrene, pyrene, polycyclic aromatic compounds (pac)

## Abstract

**Introduction:**

The ability of polycyclic aromatic compounds (PACs), most notably benzo(*a*) pyrene [B(*a*)P], to suppress antibody responses in experimental animals is well documented. Very little information, however, is available on the immunotoxicity of related PACs despite their widespread presence in the environment. Additionally, there are several weaknesses in existing immunotoxicity databases for PACs in experimental animals, limiting their applicability in quantitative risk assessment. Careful characterization of strong positive and clear negative PACs is needed in order to lay the foundation for generating robust immunotoxicity data for structurally diverse PACs that have not yet been evaluated.

**Methods:**

In the current study, adult B6C3F1/N female mice were treated daily for 28 consecutive days by oral administration of B(*a*)P to provide dose levels ranging between 2 and 150 mg/kg bodyweight/day. In addition, phenanthrene and pyrene, non-carcinogenic PACs, were tested at dose ranges between 12.5 and 800 mg/kg bodyweight/day and 3.1 and 200 mg/kg bodyweight/day, respectively. Immune assessments following PAC exposure included organ weights and immunopathology, hematology, quantification of immune cell types in the spleen, and T-dependent antibody response (TDAR) to sheep red blood cells (SRBC).

**Results:**

Benzo(*a*)pyrene exposure resulted in significant decreases in lymphoid organ weights, immune cell populations in the spleen and TDAR. The most sensitive indicator for immunotoxicity from B(*a*)P treatment was suppression of antibody responses, where an ∼75% decrease occurred at a dose level of 9 mg/kg bodyweight/day and ∼32% decrease at the lowest tested dose of 2 mg/kg bodyweight/day. Antibody suppression was associated with significant immune cell loss in the spleen; however, it was clear that the suppression of the TDAR was more sensitive than cell loss indicating that cell function impairments were involved. Phenanthrene treatment also resulted in suppression of the antibody response but only at dose levels ≥50 mg/kg bodyweight/day without significant effects on other parameters, while pyrene showed no significant immune effects.

**Conclusion:**

Suppression of the TDAR to SRBC immunization was the most sensitive immune endpoint being 33 times more sensitive than changes in liver weight, a commonly used outcome for risk assessment for PACs. Benzo(*a*)pyrene was the most potent PAC regarding suppression of humoral immunity whereas pyrene did not affect the immune responses tested. These studies lay the foundation for evaluating diverse PACs with a range of immunotoxicological potencies.

## Introduction

Polycyclic aromatic compounds (PACs) are a structurally diverse class of chemicals containing at least two fused benzene rings and encompassing polycyclic aromatic hydrocarbons (PAHs) as well as heterocyclics that contain N, O, or S within their ring structures and N-, O-, or S- substituted PAHs (Hsieh et al., 2021). They are widespread environmental contaminants with both petrogenic (e.g., petroleum products) and pyrogenic (e.g., cigarette smoke, wildfires) sources (Al Hello et al., 2023). Exposure to PACs can occur via multiple routes including inhalation of contaminated air, ingestion, or dermal contact. People are generally exposed to complex and dynamic mixtures of PACs, not individual compounds. Exposure to PAC-containing mixtures has been associated with a broad range of toxicities. In particular, many epidemiological studies suggest that exposure to PAC mixtures leads to immunotoxicity (Biró et al., 2002; Karakaya et al., 2004; Oh et al., 2006; Szczeklik et al., 1994; Winker et al., 1997; Wu et al., 2003). Therefore, methods to evaluate the risk posed by mixtures of PACs are important for protecting public health.

A relative potency factor approach is the default method used to evaluate PAC-containing mixtures (Haber et al., 2022; US EPA, 2010; US EPA, 1993). Based on the concept of dose addition, the relative potency factor approach adds potency-adjusted doses of individual PACs to estimate the risk associated with exposure to mixtures. Potency values are calculated by dividing the reference chemical dose that elicits a certain level of effect (e.g., ED50) by the individual chemical dose that causes the same level of effect. Risk evaluations of PAC-containing mixtures have typically been limited to parent PAHs and have focused almost exclusively on carcinogenicity (Haber et al., 2022). Questions remain as to whether carcinogenicity is the most sensitive endpoint for PAC mixtures, and therefore, the most health protective. Several factors contribute to increased attention on immune suppression for PAC mixtures: the role of immune suppression in carcinogenesis, involvement of the aryl hydrocarbon receptor in both cancer and immune processes, and the ability to measure functional immune suppression in shorter-term assays, as well as other key characteristics of immunotoxicants that could help define mechanisms of immunotoxicity and mode of action (Germolec et al., 2022). Considering these factors, it was posited that immunotoxicity data could be used to enhance cancer risk assessment of PAC mixtures (Zaccaria and McClure, 2013).

Benzo(a)pyrene [B(*a*)P] is the most well-studied PAC and has been identified as the reference to which other chemicals in the class are compared for generating potency factors. In addition to being classified as a Group 1 carcinogen by IARC (IARC, 2010), experimental evidence indicates that B(*a*)P displays immunotoxicity (De Jong et al., 1999; Silkworth et al., 1995; White et al., 1985), reproductive toxicity (Archibong et al., 2002; Inyang et al., 2003), developmental toxicity (Detmar et al., 2008), and neurotoxicity (Chen et al., 2012). B(*a*)P is known to decrease antigen-specific humoral immunity (De Jong et al., 1999; Dean et al., 1983; Silkworth et al., 1995; Temple et al., 1993; US EPA, 2017). Other effects reported in studies of B(*a*)P immunotoxicity included changes in total serum immunoglobulin concentrations, immune cell numbers, cytokine levels, and T and B cell proliferation.

Phenanthrene and pyrene are at the other end of the toxicity spectrum from B(*a*)P. Both are in IARC Group 3 (not classifiable as to carcinogenicity in humans) (IARC, 2010) and were given a potency factor of 0 in the unreleased draft EPA document on “Development of a Relative Potency Factor (RPF) Approach for Polycyclic Aromatic Hydrocarbon (PAH) Mixtures” (US EPA, 2010). Phenanthrene contains a bay region area, like many carcinogenic PACs, and demonstrates similarities in metabolism and detoxification to B(*a*)P, including cytochrome P450 induction (Hecht et al., 2003; Nota et al., 2009). In addition, phenanthrene induces similar mRNA transcription profiles as B(*a*)P, activating an abundance of genes that not only affect biotransformation pathways, but typical stress response pathways, such as oxidative stress and immune response genes as well (Nota et al., 2009). Previous studies showed that phenanthrene had no effect on immunosuppression following a single oral dose up to 100 mg/kg, while pyrene displayed a 30% suppression of antigen-specific T-dependent antibody production following a single oral exposure of 100 mg/kg (Silkworth et al., 1995). Phenanthrene and pyrene have not been fully characterized for other toxicities (US EPA, 2009; US EPA, 2007).

The current studies are part of a larger PAC mixtures assessment program (https://ntp.niehs.nih.gov/whatwestudy/topics/pacs) aimed at addressing several uncertainties inherent in the current default relative potency factor approach. In the studies described here, we characterized the dose-response relationship for the reference PAC, B(*a*)P, and two additional PACs with low immunotoxicity potential, phenanthrene and pyrene, that could potentially serve as negative comparators. This work lays the foundation for additional studies exploring the immunotoxicity of structurally diverse individual PACs and mixtures of PACs.

## Methods

These studies were conducted in compliance with the U.S. Food and Drug Administration Good Laboratory Practices for Nonclinical Laboratory Studies (Title 21 of the Code of Federal Regulations, Part 58) and approved by the IACUC at Burleson Research Technologies, Inc. (Morrisville, NC). The immune assessments were performed according to previous studies (Johnson et al., 2023; Watson et al., 2021) and are described below.

### Animals

Female B6C3F1/N mice were obtained from Taconic Biosciences Inc. (Hudson, NY). Following acclimation, mice were randomized (±20% of mean bodyweight) and placed in individually ventilated cages (4 mice/cage). Mice were 8–12 weeks old at the start of dosing. Mice were provided NTP-2000 diet (Ziegler Bros., Inc., Gardners, PA), *ad libitum,* in which no contaminants were found that would interfere with the conduct of the study. Bodyweights were recorded before dose administration on Day 0 and then weekly on Days 6, 13, and 20. Terminal bodyweights were recorded following euthanasia on Day 28. All animals were euthanized by CO_2_ inhalation using 100% CO_2_ introduced at 3.65 LPM into a 7.3 L chamber to displace 50% of the atmosphere per minute; confirmation of death by severing the diaphragm.

### Test chemicals, treatments, and experimental design

B(*a*)P (Lot# CR66-25–1; >99% purity) was obtained from MRI Global (Kansas City, MO), phenanthrene (Lot# 7MP2K; >98% purity) was obtained from TCI America (Portland, OR), and pyrene (Lot# 20151201; >98% purity) was obtained from Ivy Fine Chemicals (Cherry Hill, NJ). Dose formulations were prepared in corn oil by Battelle Laboratories (Columbus, OH) and analyzed for concentration using a validated gas chromatography with flame ionization detection method [B(*a*)P: r^2^ > 0.99; precision determined as relative standard deviation (RSD) ≤ 5%; accuracy determined as relative error (RE), ≤± 3%; phenanthrene: r^2^ > 0.99; RSD ≤1%; RE, ≤± 5.2%; pyrene: r^2^ > 0.99; RSD </= 1.0%; RE, </= ± 4.0%] and were within 10% of target concentrations. Corn oil formulations of PACs were prepared at concentrations to provide dose volumes of 10 mL/kg. Cyclophosphamide monohydrate (CPS; Lot# MKBS0021V; >99% purity) was obtained from Sigma Aldrich (St. Louis, MO) and was used as a positive control for studies with B(*a*)P and phenanthrene, while B(*a*)P (10 mg/kg bodyweight/day) was the positive control for the pyrene studies. The dose formulations of CPS were prepared in 0.9% saline vehicle by RTI International (RTP, NC) and analyzed for concentration using a validated high performance liquid chromatography with evaporative light scattering detection (HPLC/ELSD) method (r > 0.99; RSD ≤6.7%; RE, ≤± 10%) and were within 10% of target concentration. Samples of the dose formulations for PACs and CPS were obtained from containers used for dosing the animals and analyzed for concentration; all animal room samples were within 10% of target. Prior to study start, stability of test article in formulations were confirmed up to a minimum of 42 days.

B(*a*)P, phenanthrene and pyrene studies were conducted at different time periods, each with their own vehicle and positive control groups, using identical experimental designs and conditions. Each study consisted of two distinct cohorts of animals. The first cohort (Cohort 1; SRBC), consisting of 8 mice per treatment group, was used to evaluate the impact of chemical treatment on the T-dependent antibody response (TDAR) to sheep red blood cells (SRBC) and splenic cell immunophenotyping. A second cohort (Cohort 2; Immunopathology), also consisting of 8 mice per treatment group, was used for collection of tissues for assessment of bone marrow cellularity, histopathology of the immune system, and hematology. Vehicle (corn oil) and test articles were administered in volumes of 10 mL/kg orally, once daily for 28 days (Day 0 through Day 27). B(*a*)P treatment doses were 2, 5, 9, 19, 38, 75 and 150 mg/kg bodyweight/day, phenanthrene treatment doses were 12.5, 25, 50, 100, 200, 400, and 800 mg/kg bodyweight/day, and pyrene treatment doses were 3.1, 6.3, 12.5, 25, 50, 100, and 200 mg/kg bodyweight/day. The goal in dose selection was to cover the dose-response curve for the immune function endpoints being tested. The high dose for each chemical was expected to be below the maximum tolerated dose based on available data, while the low dose was meant to approximate the no observed effect level for general toxicity. In the B(*a*)P and phenanthrene studies, a separate group received 50 mg/kg bodyweight/day CPS via intraperitoneal (IP) injection on Days 24–27. On Day 24, CPS was administered after immunization with SRBC. In the pyrene study, a separate group received 10 mg/kg bodyweight/day B(*a*)P for 28 days (Day 0 through Day 27) to serve as a calibration point across studies. No test or control article was administered on the day of euthanasia (Day 28).

### Clinical observations, pathology, hematology, and bone marrow cell collection

Detailed clinical observations were performed for all study animals up to 2 days prior to the start of dosing and then once per week thereafter for the duration of treatment. Signs of toxicity, including onset, degree, and duration, were documented. Observations included evaluation of skin and fur, eyes and mucous membranes, respiratory and circulatory effects; autonomic effects (e.g., salivation), central nervous system effects (e.g., tremors and convulsions, changes in the level of activity, gait and posture, reactivity to handling), and behavioral changes (e.g., self-mutilation, walking backwards).

On Day 28, all surviving mice in Cohort two were anesthetized by CO_2_ inhalation and blood was collected by the retro-orbital route. The first drop or two of blood was discarded prior to collection into a K_2_EDTA blood collection tube to minimize the risk of micro-clots in the sample. The blood was shipped on ice packs on the day of blood collection for analysis (complete blood counts with white blood cell differential and reticulocyte counts) to Antech-GLP (Morrisville, NC). Blood samples were analyzed using an Advia 120 hematology analyzer with associated V.6.3.2-M software (Siemens Medical Solutions United States, Inc., Malvern, PA). The following hematologic parameters were assessed: erythrocyte count, hematocrit, hemoglobin concentration, mean cell volume, mean cell hemoglobin, mean cell hemoglobin concentration, platelet count, reticulocyte count, leukocyte count, and white blood cell differential.

Following completion of blood collection, mice were immediately euthanized with CO_2_ and selected tissues for histopathology were collected. Gross observations were documented and the spleen, thymus, liver, right kidney with adrenal gland, ovaries (single weight for the pair), and lungs were weighed. The spleen, thymus, mesenteric and popliteal lymph nodes, left femur with bone marrow, lung (including bronchial-associated lymphoid tissue [BALT]), right kidney with adrenal gland, left ovary, and liver (median, caudate, and right lobes) were preserved in 10% neutral buffered formalin (NBF). Other organs and tissues showing gross lesions were preserved in 10% NBF. The spleen, thymus, lymph nodes, BALT, and bone marrow were examined using the enhanced histopathology method (Elmore, 2012). Enhanced histopathology is a systematic approach used to characterize, both qualitatively and semi-quantitatively, immuno-modulatory effects within lymphoid organs after exposure to potentially immunotoxic or immunomodulatory compounds. Bone marrow cells from the right femur were removed, collected and total cell numbers determined.

### Antibody tests

SRBC in Alsever’s (Colorado Serum Company, Denver, CO) were washed 3 times in phosphate buffered saline (PBS) and resuspended to a final concentration of 3.75 × 10^8^ SRBC/mL. Mice were intravenously immunized via the tail vein with 0.2 mL of the 3.75 × 10^8^ SRBC/mL (7.5 × 10^7^ SRBC/mouse) of the preparation on Day 24. Four days after immunization, animals were euthanized with CO_2_ and weighed. A maximum amount of blood was collected by cardiac puncture or from the inferior vena cava of each animal into a serum separator collection tube. The spleen and thymus were removed, and weight recorded. Blood was collected into tubes and allowed to clot at room temperature for 30–60 min. The tubes were centrifuged at approximately 1300xg at room temperature for approximately 10 min. Serum for each animal was then collected and stored at ≤ -70°C until evaluated for anti-SRBC IgM serum antibodies.

The antibody forming cell (AFC) response to SRBC, a T-dependent response, was used to assess the impact of PACs on humoral immunity. Spleens were processed to single cell suspensions in HBSS + HEPES and cell concentration and viability were determined. Spleen cells (1:30 and 1:120 dilution in 100 μL) and SRBC (25 μL of ∼50% suspension in HBSS) were added to 500 μL of molten agar media (at 44°C ± 1°C) and mixed with 25 μL of guinea pig complement (1/3 dilution of stock in 1 mL of HBSS with 0.1 mL of 50% SRBC suspension; Cedarlane Laboratories, Burlington, NC) in duplicate tubes. Resulting suspensions were poured onto the center of a Petri dish (in duplicate) and covered with glass. The agar was allowed to solidify prior to being placed in an incubator set to maintain 37°C for at least 3 hours and then AFC plaques were enumerated. The number of plaques were expressed per million spleen cells and per spleen.

Serum samples from individual animals were evaluated for IgM antibody titers to SRBC using a commercial ELISA kit (Life Diagnostics, St. Petersburg, FL). Briefly, diluted test samples and standards were added to microwells and incubated for 45 min. The wells were washed, and horse radish peroxidase-conjugated anti-mouse IgM added to the wells. The microplate was incubated at room temperature for 45 min, the wells washed, and the substrate solution added. Color development was stopped after 20 min by addition of the stop solution. The optical density was determined spectrophotometrically at 450 nm using a Spectramax 340 (Molecular Devices, Sunnyvale, CA). All samples and standards were run in duplicate and data analysis was performed using Softmax Pro^®^ version 2.2.1 software (Molecular Devices).

### Immunophenotyping

Spleens from the SRBC cohort were subjected to ammonium chloride RBC lysis. The resulting mononuclear cells were re-suspended in RPMI with 5% FBS to 2.5 × 10^6^ cells/mL; and 100 μL aliquots containing 2.5 × 10^5^ cells were added to cluster tubes and the cells pelleted. The cells were then re-suspended in 50 µL stain buffer (PBS/2% BSA/0.1% NaN3) and incubated for 5–30 min on ice after addition of Fc Block solution (BioLegend, San Diego, CA). Following the blocking step, 50 μL of antibody (all BioLegend) cocktails containing antibodies to: (1) Anti-Mouse CD3, Anti-Mouse CD161a, Anti-Mouse CD45, and Anti-Mouse CD45RA; (2) Anti-Mouse CD8a, Anti-Mouse CD3, Anti-Mouse CD45, and Anti-Mouse CD4; or (3) Anti-Mouse CD11b, Anti-Mouse CD11c, Anti-Mouse Ly6G, and Anti-Mouse NKp46 were added to the appropriate tubes. Control tubes contained cells only, cells with a single antibody from the list above, or cells with a single isotype control antibody. The tubes were incubated on ice, protected from light, for 20–50 min. Following incubation, the samples were fixed using 2% paraformaldehyde for at least 30 min, followed by centrifugation and re-suspension in fresh stain buffer. The samples were then stored at 2-8°C, protected from light, until analyzed on an Accuri C6 flow cytometer using CFlow Plus v 1.0.264.15 (BD Biosciences). In all cases, a minimum of 20,000 events/sample was acquired.

Lymphocyte gating was performed on CD45^+^ populations. The following lymphocyte subsets were identified; T cells (CD3^+^CD45RA-), B cells (CD3^−^CD45RA+), NK cells (CD3^−^CD161a+), T-helper cells (CD3^+^CD4^+^), and T-cytotoxic cells (CD3^+^CD8^+^). Myeloid cells were gated based on being positive for CD11b with low-to-mid intensity staining for CD11c. Myeloid populations were differentiated from NK cells based on lack of NKp46 expression. Further differentiation was based on expression of Ly6G with positive cells being neutrophils and negative cells differentiated using SSC into monocytes/macrophages with low granularity and eosinophils with high granularity.

### Data collection and statistical analysis

Data were collected into Provantis v9.2.3 (Instem, Philadelphia, PA) and calculation of endpoints was performed within this validated system. Results are presented as mean ± SEM. Bodyweight and organ weight data, which typically exhibit a normal distribution, were analyzed using a parametric multiple comparison procedure. If a significant trend was detected at p ≤ 0.01, Williams’ test was used (Williams, 1986); if the trend was not significant, Dunnett’s test was used (Dunnett, 1955). Positive control bodyweight and organ weight data were compared to the vehicle control group using a standard t-test. Data for other endpoints were analyzed using a non-parametric multiple comparison procedure. If a significant trend was observed, Shirley’s test was used (Shirley, 1977); if the trend was not significant, Dunn’s test was used (Dunn, 1964). Positive control group data were compared to the vehicle control group using the Wilcoxon rank-sum test (Mann and Whitney, 1947). Data that were different from control at p ≤ 0.05 were considered statistically significant. Extreme values were identified by an outlier test (Dixon and Massey, 1957). All flagged outliers were examined by NIEHS personnel, and statistical outliers that were biologically implausible were eliminated from the final analyses.

Histopathology data were analyzed using a Cochran-Armitage trend test and Fisher Exact pairwise tests. These tests were one-sided.

### Benchmark dose analyses of organ weights, immune cell populations, and antibody production

Dose response modeling was performed using BMDS Online (US EPA, 2024) to derive a benchmark dose (BMD) for organ weights and immune endpoints for each PAC. The benchmark reference used for the modeling was one standard deviation (1SD) of the control group data, as it is a commonly used reference for safety and risk assessment. All endpoints were modeled as continuous data using the models provided in BMDS Online including the following.

- Exponential M3 and M5
- Hill
- Polynomial two and 3
- Power
- Linear

Distributions for the endpoints were considered normal and the variance was set as constant or non-constant based on the data set and recommendations of BMDS Online. When no viable model (questionable and unusable output) fit the raw data, dose levels were transformed by the natural logarithm of dose +1 (constant to facilitate transformation of the vehicle group) and modeling was repeated. If no viable model was identified for the original or transformed data, no BMD or BMD_L_ (lower limit of BMD) were presented. When more than one model was viable for a data set, BMDS Online recommended the best fit model based on the lowest BMD_L_ or Akaike information criterion (AIC) for each data set the best fit model was used to report the BMD and BMD_L_. A relative change of 1xSD from control was used to provide context for the BMD calculation.

## Results

Summary findings relevant for evaluating immune toxicity are presented below. All study findings (including individual animal data) are available at the NTP Chemical Effects in Biological Systems (CEBS) database https://doi.org/10.22427/NTP-DATA-500-005-004-000-4.

### Clinical observations and bodyweights

Several mice in the 800 mg/kg bodyweight/day phenanthrene group exhibited overt toxicity within the first 2 days of dosing and the group was subsequently removed from the study due to excessive toxicity. All remaining mice dosed with 800 mg/kg bodyweight/day were humanely euthanized using CO_2_. Several mice in the remaining phenanthrene treatment groups showed labored breathing, hunched back and/or piloerection within the first 2 weeks of commencing dosing and subsequently were euthanized by CO_2_. These effects were not dose related and necropsy results indicated that they were the result of gavage trauma. There was no other clinical evidence of significant systemic toxicity in any of the other phenanthrene treated mice nor in any of the B(*a*)P or pyrene treated groups (CEBS Summary Tables I05). While there were no bodyweight changes related to phenanthrene or pyrene treatment, there were statistically significant treatment-related changes in bodyweight gains and bodyweights following B(*a*)P treatment (CEBS Summary Tables I04 and I04G). Bodyweight showed a significant negative trend with increasing dose of B(*a*)P on Days 20 and 28 and was significantly reduced in the 150 mg/kg bodyweight/day treatment group relative to the vehicle control group. However, the decrease in bodyweight was ∼10% compared to the vehicle control group and was unlikely to be responsible for the observed effects of B(*a*)P on the immune system.

### Organ weights

At necropsy on Day 28 the spleen, thymus, liver, right kidney with adrenal gland, ovaries (single weight for the pair) were removed and weighed. In addition, popliteal and mesenteric lymph nodes were collected and examined histologically. There were significant decreases in absolute spleen weights in mice treated with 75 and 150 mg/kg bodyweight/day B(*a*)P, thymus weights [≥19 mg/kg bodyweight/day B(*a*)P] and thymus to bodyweight ratios [≥38 mg/kg bodyweight/day B(*a*)P] relative to the vehicle control group (Table 1; CEBS Summary Tables PA06). Absolute liver weights were increased only in mice treated with 150 mg/kg bodyweight/day B(*a*)P while liver to bodyweight ratios were significantly increased in groups administered doses of ≥19 mg/kg bodyweight/day (Table 1). There were also significant decreases in the absolute combined kidney and adrenal gland weight as well as relative and absolute ovary weights in mice treated with 150 mg/kg bodyweight/day B(*a*)P (CEBS Summary Table PA06). Although there were no significant effects on spleen or thymus weights in phenanthrene treated groups, liver weight (≥200 mg/kg bodyweight/day phenanthrene) and liver to bodyweight ratios (≥50 mg/kg bodyweight/day phenanthrene) were increased relative to the vehicle control group (Table 1). Relative spleen weight was significantly increased in mice exposed to 25 mg/kg bodyweight/day pyrene, although this isolated change is likely not biologically meaningful (Table 1). There was a negative trend for relative kidney weight with a significant reduction in mice treated with 200 mg/kg bodyweight/day pyrene (CEBS Summary Table PA06). Statistically significant changes in organ weights were observed in mice treated with CPS including decreases in the thymus and spleen weights and a slight but statistically significant increase in the liver weights in the studies conducted for B(*a*)P and phenanthrene. The positive control for the pyrene study, 10 mg/kg bodyweight/day B(*a*)P, did not result in any changes in organ weights, as expected for this dose level based on the B(*a*)P studies.

**TABLE 1:** Spleen, thymus and liver weights in female B6C3F1/N mice treated orally with benzo(a)pyrene, phenanthrene, or pyrene (Cohort 2).

	Benzo(a)pyrene (mg/kg bodyweight/day)								CPS 50 mg/kg body weight/day
	0	2	5	9	19	38	75	150	
Terminal BW	25.0 ± 0.9^↓↓^	24.7 ± 1.0	24.5 ± 0.7	25.0 ± 1.0	24.1 ± 0.8	23.9 ± 0.8	23.0 ± 0.3	22.5 ± 0.5*	25.4 ± 1.0
Spleen (mg)	0.068 ± 0.004^↓↓^	0.067 ± 0.003	0.067 ± 0.004	0.064 ± 0.003	0.067 ± 0.002	0.064 ± 0.002	0.057 ± 0.003*	0.053 ± 0.003**	0.049 ± 0.002**
Spleen:BW Ratio	2.73 ± 0.15^↓^	2.73 ± 0.13	2.76 ± 0.17	2.56 ± 0.11	2.78 ± 0.05	2.69 ± 0.09	2.48 ± 0.11	2.35 ± 0.10	1.94 ± 0.08**
Thymus (mg)	0.057 ± 0.002^↓↓^	0.054 ± 0.004	0.056 ± 0.002	0.060 ± 0.003	0.048 ± 0.003*	0.041 ± 0.003**	0.040 ± 0.002**	0.024 ± 0.002**	0.018 ± 0.001**
Thymus:BW Ratio	2.29 ± 0.09^↓↓^	2.19 ± 0.13	2.29 ± 0.09	2.40 ± 0.12	2.01 ± 0.15	1.71 ± 0.12**	1.71 ± 0.07**	1.06 ± 0.10**	0.74 ± 0.05**
Liver Weight (g)	1.11 ± 0.05^↑↑^	1.06 ± 0.05	1.10 ± 0.04	1.14 ± 0.05	1.20 ± 0.06	1.20 ± 0.04	1.13 ± 0.03	1.40 ± 0.04**	1.23 ± 0.03
Liver:BW Ratio	44.56 ± 0.84^↑↑^	42.78 ± 0.79	45.12 ± 1.18	45.53 ± 0.79	49.49 ± 1.28**	50.37 ± 1.27**	48.85 ± 0.83**	61.99 ± 0.78**	48.50 ± 1.25*

	Phenanthrene (mg/kg bodyweight/day)								CPS 50 mg/kg bodyweight/day
	0	12.5	25	50	100	200	400	800^a^	
Terminal BW	23.7 ± 1.0	22.6 ± 0.7	23.5 ± 0.6	23.7 ± 0.8	22.9 ± 0.9	23.1 ± 0.7	22.8 ± 0.7	N/A	23.7 ± 0.6
Spleen (mg)	0.069 ± 0.003	0.063 ± 0.003	0.069 ± 0.003	0.076 ± 0.005	0.065 ± 0.006	0.070 ± 0.002	0.063 ± 0.005	N/A	0.051 ± 0.003**
Spleen:BW Ratio	2.93 ± 0.07	2.75 ± 0.07	2.93 ± 0.13	3.20 ± 0.18	2.87 ± 0.25	3.01 ± 0.11	2.80 ± 0.23	N/A	2.12 ± 0.09**
Thymus (mg)	0.060 ± 0.002	0.059 ± 0.003	0.065 ± 0.005	0.061 ± 0.006	0.061 ± 0.006	0.061 ± 0.003	0.056 ± 0.006	N/A	0.020 ± 0.002**
Thymus:BW Ratio	2.55 ± 0.09	2.57 ± 0.12	2.76 ± 0.20	2.53 ± 0.20	2.68 ± 0.29	2.54 ± 0.09	2.47 ± 0.23	N/A	0.84 ± 0.07**
Liver Weight (g)	1.10 ± 0.06^↑↑^	1.01 ± 0.02	1.11 ± 0.02	1.22 ± 0.06	1.19 ± 0.04	1.38 ± 0.05**	1.45 ± 0.05**	N/A	1.20 ± 0.06
Liver:BW Ratio	46.41 ± 1.25^↑↑^	44.91 ± 0.85	47.1 ± 0.92	51.27 ± 1.53*	52.38 ± 1.97**	58.42 ± 1.58**	63.71 ± 1.00**	N/A	50.27 ± 1.44

	Pyrene (mg/kg bodyweight/day)								B(*a*)P 10 mg/kg bodyweight/day
	0	3.1	6.3	12.5	25	50	100	200	
Terminal BW	23.8 ± 0.9	23.7 ± 0.6	24.0 ± 0.8	24.8 ± 0.9	24.1 ± 0.4	24.2 ± 1.0	24.7 ± 0.9	24.3 ± 0.8	25.1 ± 1.3
Spleen (mg)	0.062 ± 0.004	0.070 ± 0.003	0.070 ± 0.004	0.070 ± 0.005	0.076 ± 0.002	0.076 ± 0.007	0.067 ± 0.004	0.072 ± 0.006	0.073 ± 0.004
Spleen:BW Ratio	2.60 ± 0.14	2.96 ± 0.10	2.92 ± 0.10	2.82 ± 0.15	3.16 ± 0.07*	3.13 ± 0.22	2.70 ± 0.12	2.95 ± 0.19	2.93 ± 0.07
Thymus (mg)	0.047 ± 0.003	0.049 ± 0.001	0.044 ± 0.004	0.046 ± 0.002	0.049 ± 0.002	0.045 ± 0.002	0.044 ± 0.005	0.049 ± 0.003	0.047 ± 0.005
Thymus:BW Ratio	1.99 ± 0.15	2.04 ± 0.04	1.83 ± 0.14	1.90 ± 0.12	2.02 ± 0.10	1.86 ± 0.10	1.76 ± 0.19	2.03 ± 0.13	1.89 ± 0.18
Liver Weight (g)	1.04 ± 0.03	1.03 ± 0.03	1.06 ± 0.04	1.08 ± 0.06	1.07 ± 0.03	1.10 ± 0.06	1.05 ± 0.06	1.13 ± 0.05	1.13 ± 0.07
Liver:BW Ratio	43.98 ± 1.05	43.53 ± 0.81	44.00 ± 0.75	43.30 ± 1.22	44.35 ± 0.91	45.39 ± 1.20	42.44 ± 0.97	46.66 ± 0.95	45.07 ± 1.08

Data are presented as mean ± standard error of the mean. CPS-Cyclophosphamide; B(*a*)P-benzo(a)pyrene; N/A–not applicable.

^a^ Group removed due to overt toxicity. See CEBS, Summary Table PA06 for group sizes and CEBS, Summary Table I02 for unscheduled deaths. Increasing (^↑↑^p < 0.01) or decreasing (^↓^p < 0.05 or ^↓↓^p < 0.01) trend with increasing dose of PAC., Significantly different from the vehicle control group at *p < 0.05 or **p < 0.01.

### Histopathology

Histopathological findings are provided in CEBS Summary Tables PA02, PA03, PA05, PA10, and PA18. B(*a*)P treatment resulted in several statistically significant histopathological changes in the thymus including minimal to moderately decreased cortical area [≥38 mg/kg bodyweight/day B(*a*)P], minimally to moderately decreased cortical lymphocyte numbers [≥38 mg/kg bodyweight/day B(*a*)P], minimally to mildly decreased medullary area [150 mg/kg bodyweight/day B(*a*)P], and minimally decreased medullary lymphocyte numbers [150 mg/kg bodyweight/day B(*a*)P] (Table 2). In addition, animals exposed to B(*a*)P showed minimal to moderate decreases in the thymic cortex:medulla ratio [≥38 mg/kg bodyweight/day B(*a*)P] and minimally increased apoptotic cells in the thymic cortex and medulla [150 mg/kg bodyweight/day B(*a*)P] (Table 2). Histological changes in the spleen of B(*a*)P treated groups included minimally to mildly decreased periarteriolar lymphoid sheath lymphocyte numbers [150 mg/kg bodyweight/day B(*a*)P] and a mild to moderate decrease in the number and size of follicular germinal centers [≥38 mg/kg bodyweight/day B(*a*)P] (Table 2). Histopathologic changes were also identified in the paracortex of the mesenteric lymph nodes associated with B(*a*)P treatment. These included minimally to moderately increased area and increased lymphocyte number in all groups treated with ≥38 mg/kg bodyweight/day B(*a*)P (Table 2). There were no attributable effects on popliteal lymph nodes, bone marrow, or BALT in B(*a*)P treated mice. There was a significant mild to moderate decrease in the corpus luteum numbers in the ovaries of mice treated with ≥75 mg/kg bodyweight/day B(*a*)P. Histopathological changes observed in mice treated with phenanthrene or pyrene were sporadic and not considered to be related to treatment. Histopathological examination was not conducted in CPS treated mice.

**TABLE 2:** Incidence and severity of histopathological changes in female B6C3F1/N mice treated orally with benzo(a)pyrene.

	Benzo(*a*)pyrene (mg/kg bodyweight/day)							
	0	2	5	9	19	38	75	150
Thymus								
Cortex/Medulla Ratio - Decreased	0^↑↑^	1 [2.0]	0	2 [1.5]	3 [1.7]	6 [1.5]**	7 [1.6]**	8 [2.6]**
Cortex - Decreased Area	0^↑↑^	0	0	2 [1.5]	3 [1.7]	6 [1.5]**	7 [1.7]**	7 [3.0]**
Medulla - Decreased Area	0^↑↑^	0	0	0	2 [1.0]	2 [1.0]	2 [1.0]	6 [1.2]**
Cortex - Decreased Lymphocyte Number	0^↑↑^	0	0	2 [1.5]	3 [1.7]	6 [1.5]**	7 [1.7]**	8 [2.9]**
Medulla - Decreased Lymphocyte Number	0^↑↑^	0	0	1 [2.0]	2 [1.0]	2 [1.0]	3 [1.0]	8 [1.1]**
Cortex - Increased Apoptotic Lymphocyte Number	0^↑↑^	0	0	0	1 [1.0]	2 [1.0]	1 [1.0]	8 [1.0]**
Medulla - Increased Apoptotic Lymphocyte Number	0^↑↑^	0	0	0	0	0	1 [1.0]	7 [1.0]**
Spleen								
Periarteriolar Lymphoid Sheath - Decreased Lymphocyte Number	0^↑↑^	0	0	0	2 [1.0]	3 [1.0]	3 [1.3]	4 [1.3]*
Germinal Center Follicle - Decreased Number	0^↑↑^	1 [2.0]	1 [1.0]	1 [1.0]	3 [2.0]	5 [2.6]*	8 [1.8]**	8 [2.3]**
Mesenteric Lymph Node								
Paracortex - Increased Area	0^↑↑^	0	1 [1.0]	2 [1.5]	2 [2.0]	8 [1.8]**	5 [2.8]*	7 [2.0]**
Paracortex - Increased Lymphocyte Number	0^↑↑^	0	1 [1.0]	2 [1.5]	2 [2.0]	8 [1.8]**	5 [2.6]*	7 [2.0]**

N = 8 per treatment group.

Data are presented as number of animals affected [severity grade]; Severity grade: 1 – minimal, 2 – mild, 3 – moderate, 4 – marked.

^↑↑^Significant increasing trend with increasing dose of B(*a*)P at p < 0.01.

Significantly different from the vehicle control group at *p < 0.05 or **p < 0.01.

### Hematology

Hematological profiles and white blood cell differentials were determined on Day 28 following oral exposure to B(*a*)P (Table 3), phenanthrene (Table 4), or pyrene (Table 5). Significant decreases were observed in most erythrocytic parameters including erythrocyte counts (≥19 mg/kg bodyweight/day), hemoglobin and hematocrit values (≥38 mg/kg bodyweight/day), and mean cell hemoglobin concentration in mice treated with 150 mg/kg bodyweight/day B(*a*)P (Table 3). Mean cell hemoglobin and mean cell volumes (≥19 mg/kg bodyweight/day) and platelet counts (150 mg/kg bodyweight/day) were increased by B(*a*)P treatment. Increased platelet counts were observed in mice treated with 400 mg/kg bodyweight/day phenanthrene (Table 4). Mice exposed to ≥100 mg/kg bodyweight/day pyrene showed significantly decreased mean cell hemoglobin relative to the vehicle control group (Table 5).

**TABLE 3:** Hematology analyses in blood of female B6C3F1/N mice treated orally with benzo(*a*)pyrene.

	Benzo(a)pyrene (mg/kg bodyweight/day)								CPS
	0	2	5	9	19	38	75	150	50 mg/kg bodyweight/day^2^
Erythrocyte (x10^6^)	10.353 ± 0.092^↓↓^	10.376 ± 0.085	10.135 ± 0.109	10.188 ± 0.082	9.825 ± 0.157**	9.459 ± 0.143**	9.700 ± 0.213**	8.994 ± 0.072**	9.244 ± 0.098**
Hemoglobin (g/dL)	15.43 ± 0.10^↓↓^	15.29 ± 0.13	15.05 ± 0.16	15.36 ± 0.18	15.06 ± 0.21	14.29 ± 0.48*	14.98 ± 0.28*	13.73 ± 0.10**	13.88 ± 0.12**
Hematocrit (%)	50.81 ± 0.49^↓↓^	50.78 ± 0.40	49.91 ± 0.54	50.54 ± 0.46	49.34 ± 0.84	48.28 ± 0.55*	48.91 ± 1.03*	46.78 ± 0.36**	46.04 ± 0.43**
MCV (fL)	49.09 ± 0.23^↑↑^	48.95 ± 0.24	49.25 ± 0.19	49.59 ± 0.2	50.23 ± 0.14**	51.04 ± 0.27**	50.46 ± 0.21**	51.99 ± 0.18**	49.79 ± 0.25
MCH (pg)	14.91 ± 0.12^↑↑^	14.74 ± 0.06	14.86 ± 0.07	15.08 ± 0.10	15.34 ± 0.08*	15.10 ± 0.41*	15.44 ± 0.13*	15.26 ± 0.06*	15.04 ± 0.09
MCHC (g/dL)	30.36 ± 0.26	30.10 ± 0.16	30.18 ± 0.14	30.40 ± 0.12	30.53 ± 0.21	29.59 ± 0.86	30.59 ± 0.18	29.39 ± 0.06*	30.18 ± 0.15
Platelets (x10^3^)	972.1 ± 36.8^↑↑^	1036.1 ± 28.8	1013.9 ± 22.8	990.8 ± 29.6	1108.1 ± 44.4	1027.1 ± 43.5	940.1 ± 81.0	1639.1 ± 62.4**	1229.5 ± 31.6**
Leukocyte (x10^3^)	4.63 ± 0.41^↓↓^	4.52 ± 0.38	4.45 ± 0.28	4.65 ± 0.41	4.86 ± 0.38	3.95 ± 0.59	4.06 ± 0.33	3.16 ± 0.33*	1.44 ± 0.20**
Reticulocytes (x10^3^)	297.96 ± 11.48	307.3 ± 14.91	315.46 ± 19.73	287.95 ± 11.27	317.91 ± 18.97	289.81 ± 22.85	310.84 ± 15.71	256.31 ± 14.09	75.23 ± 9.69**
Reticulocytes (%)	2.878 ± 0.104	2.964 ± 0.148	3.113 ± 0.192	2.829 ± 0.107	3.243 ± 0.209	3.049 ± 0.205	3.199 ± 0.125	2.851 ± 0.157	0.814 ± 0.106**

Data are presented as mean ± standard error of the mean. CPS-Cyclophosphamide; MCV, mean corpuscular volume; MCH, mean corpuscular hemoglobin; MCHC, mean corpuscular hemoglobin concentration. See CEBS, Summary Table M04 for group sizes and CEBS, Summary Table I02 for unscheduled deaths. Increasing (^↑↑^p < 0.01) or decreasing (^↓↓^p < 0.01) trend with increasing dose of PAC., Significantly different from the vehicle control group at *p < 0.05 or **p < 0.01.

**TABLE 4:** Hematology analyses in blood of female B6C3F1/N mice treated orally with phenanthrene.

	Phenanthrene (mg/kg bodyweight/day)								CPS
	0	12.5	25	50	100	200	400	800^a^	50 mg/kg bodyweight/day^2^
Erythrocyte (x10^6^)	10.252 ± 0.149	10.344 ± 0.128	10.402 ± 0.113	10.494 ± 0.249	10.348 ± 0.076	10.346 ± 0.070	10.195 ± 0.130	N/A	8.881 ± 0.084**
Hemoglobin (g/dL)	15.35 ± 0.21	15.60 ± 0.16	15.45 ± 0.20	15.84 ± 0.30	15.60 ± 0.16	15.57 ± 0.11	15.19 ± 0.21	N/A	13.46 ± 0.13**
Hematocrit (%)	50.50 ± 0.85	51.53 ± 0.68	50.83 ± 0.47	52.26 ± 1.03	50.82 ± 0.42	50.93 ± 0.36	50.36 ± 0.69	N/A	44.45 ± 0.47**
MCV (fL)	49.25 ± 0.23	49.80 ± 0.27	48.87 ± 0.32	49.78 ± 0.40	49.14 ± 0.29	49.21 ± 0.28	49.40 ± 0.25	N/A	50.04 ± 0.24*
MCH (pg)	14.98 ± 0.06	15.06 ± 0.11	14.87 ± 0.16	15.10 ± 0.14	15.06 ± 0.10	15.06 ± 0.08	14.90 ± 0.06	N/A	15.16 ± 0.05*
MCHC (g/dL)	30.40 ± 0.18	30.29 ± 0.23	30.45 ± 0.23	30.34 ± 0.09	30.68 ± 0.39	30.57 ± 0.19	30.15 ± 0.13	N/A	30.26 ± 0.11
Platelets (x10^3^)	1001.2 ± 68.5^↑↑^	992.3 ± 24.3	1012.7 ± 53.9	946.4 ± 62.1	1063.4 ± 44.8	1076.4 ± 72.8	1298.8 ± 48.5*	N/A	1237.8 ± 36.2**
Leukocyte (x10^3^)	5.01 ± 0.60	3.96 ± 0.45	4.42 ± 0.73	4.97 ± 0.35	4.70 ± 0.51	4.32 ± 0.33	4.48 ± 0.66	N/A	1.46 ± 0.14**
Reticulocytes (x10^3^)	327.03 ± 24.11	340.46 ± 13.67	327.83 ± 15.02	389.36 ± 20.16	408.16 ± 38.36	327.24 ± 36.50	320.46 ± 15.78	N/A	82.85 ± 16.88**
Reticulocytes (%)	3.200 ± 0.263	3.287 ± 0.105	3.152 ± 0.137	3.706 ± 0.141	3.946 ± 0.370	3.157 ± 0.341	3.143 ± 0.148	N/A	0.934 ± 0.192**

Data are presented as mean ± standard error of the mean. CPS-Cyclophosphamide; MCV, mean corpuscular volume; MCH, mean corpuscular hemoglobin; MCHC, mean corpuscular hemoglobin concentration; N/A–not applicable.

^a^ Group removed due to overt toxicity. See CEBS, Summary Table M04 for group sizes and CEBS, Summary Table I02 for unscheduled deaths. Increasing trend with increasing dose of PAC, at

^↑↑^p < 0.01. Significantly different from the vehicle control group at *p < 0.05 or **p < 0.01.

**TABLE 5:** Hematology analyses in blood of female B6C3F1/N mice treated orally with pyrene.

	Pyrene (mg/kg bodyweight/day)								B(*a*)P
	0	3.1	6.3	12.5	25	50	100	200	10 mg/kg bodyweight/day
Erythrocyte (x10^6^)	10.230 ± 0.228	10.225 ± 0.196	10.389 ± 0.089	10.405 ± 0.092	10.193 ± 0.108	10.281 ± 0.091	10.445 ± 0.131	10.336 ± 0.137	10.406 ± 0.061
Hemoglobin (g/dL)	15.61 ± 0.18^↓^	15.38 ± 0.11	15.59 ± 0.09	15.51 ± 0.14	15.25 ± 0.15	15.31 ± 0.14	15.28 ± 0.17	15.24 ± 0.16	15.64 ± 0.11
Hematocrit (%)	52.36 ± 1.28	52.30 ± 1.08	53.39 ± 0.66	53.06 ± 0.56	52.33 ± 0.67	52.41 ± 0.51	52.70 ± 0.73	52.21 ± 0.70	53.78 ± 0.34
MCV (fL)	51.16 ± 0.37^↓^	51.16 ± 0.26	51.39 ± 0.47	51.01 ± 0.32	51.34 ± 0.24	50.99 ± 0.21	50.46 ± 0.22	50.54 ± 0.32	51.68 ± 0.17
MCH (pg)	15.30 ± 0.26^↓↓^	15.10 ± 0.23	15.01 ± 0.09	14.89 ± 0.07	14.95 ± 0.07	14.89 ± 0.06	14.60 ± 0.07**	14.77 ± 0.09**	14.99 ± 0.06
MCHC (g/dL)	29.90 ± 0.62	29.50 ± 0.51	29.25 ± 0.28	29.21 ± 0.15	29.14 ± 0.18	29.19 ± 0.12	28.95 ± 0.19	29.20 ± 0.14	29.05 ± 0.0.09
Platelets (x10^3^)	872.9 ± 34.3	833.1 ± 48.2	885.0 ± 59.3	791.9 ± 82.2	882.8 ± 49.8	831.0 ± 100.7	812.6 ± 83.8	833.6 ± 71.7	782.0 ± 115.9
Leukocyte (x10^3^)	6.227 ± 0.904	6.051 ± 0.859	6.344 ± 0.865	5.718 ± 0.543	6.111 ± 0.831	6.676 ± 0.618	5.229 ± 0.492	6.803 ± 0.880	6.741 ± 0.347
Reticulocytes (x10^3^)	259.64 ± 13.97	293.58 ± 11.83	291.46 ± 12.97	287.39 ± 11.70	296.08 ± 15.04	283.46 ± 15.9	270.60 ± 14.04	294.66 ± 17.74	284.58 ± 20.14
Reticulocytes (%)	2.537 ± 0.121	2.880 ± 0.132	2.808 ± 0.136	2.761 ± 0.112	2.909 ± 0.156	2.758 ± 0.160	2.586 ± 0.116	2.844 ± 0.146	2.740 ± 0.206

Data are presented as mean ± standard error of the mean. CPS-Cyclophosphamide; B(*a*)P-Benzo(*a*)pyrene; MCV, mean corpuscular volume; MCH, mean corpuscular hemoglobin; MCHC, mean corpuscular hemoglobin concentration. See CEBS, Summary Table M04 for group sizes and CEBS, Summary Table I02 for unscheduled deaths. Decreasing (^↓^p < 0.05 or ^↓↓^p < 0.01) trend with increasing dose of PAC., Significantly different from the vehicle control group at **p < 0.01.

Leukocyte counts were decreased in mice exposed to 150 mg/kg bodyweight/day B(*a*)P as were the number of lymphocytes (Table 6). Eosinophil counts and relative percentage were decreased in mice exposed to ≥75 mg/kg bodyweight/day B(*a*)P. There were no significant changes in white blood cell differentials following phenanthrene (CEBS Summary Table M03) or pyrene (CEBS Summary Table M03) exposure. CPS treatment reduced most hematological endpoints as well as produced leukocytopenia (Tables 6, 7).

**TABLE 6:** White blood cell counts and differentials in blood of female B6C3F1/N mice treated orally with benzo(a)pyrene.

	Benzo(*a*)pyrene (mg/kg bodyweight/day)								CPS
	0	2	5	9	19	38	75	150	50 mg/kg bodyweight/day
Leukocytes (K/μL)	4.633 ± 0.407^↓↓^	4.518 ± 0.377	4.450 ± 0.283	4.648 ± 0.411	4.860 ± 0.377	3.946 ± 0.587	4.056 ± 0.326	3.159 ± 0.329*	1.435 ± 0.204**
Lymphocytes (K/μL)	3.906 ± 0.329^↓↓^	3.844 ± 0.296	3.845 ± 0.213	4.018 ± 0.342	4.214 ± 0.317	3.331 ± 0.480	3.546 ± 0.290	2.463 ± 0.259**	1.280 ± 0.182**
Lymphocytes (%)	84.61 ± 0.89	85.38 ± 1.01	86.79 ± 1.05	86.69 ± 0.79	86.85 ± 0.70	84.89 ± 1.18	87.41 ± 0.72	77.96 ± 0.97	89.16 ± 0.77**
Neutrophils (K/μL)	0.524 ± 0.063	0.481 ± 0.082	0.418 ± 0.063	0.451 ± 0.059	0.465 ± 0.047	0.450 ± 0.095	0.376 ± 0.035	0.575 ± 0.064	0.101 ± 0.017**
Neutrophils (%)	11.20 ± 0.71	10.39 ± 0.91	9.09 ± 0.83	9.64 ± 0.60	9.54 ± 0.47	10.94 ± 1.01	9.33 ± 0.62	18.18 ± 0.91	7.11 ± 0.55**
Monocytes (K/μL)	0.088 ± 0.013	0.081 ± 0.012	0.084 ± 0.015	0.074 ± 0.012	0.066 ± 0.007	0.066 ± 0.014	0.068 ± 0.008	0.060 ± 0.008	0.018 ± 0.005**
Monocytes (%)	1.80 ± 0.17	1.71 ± 0.14	1.81 ± 0.24	1.60 ± 0.18	1.43 ± 0.11	1.61 ± 0.19	1.63 ± 0.12	1.89 ± 0.20	1.24 ± 0.16*
Eosinophils (K/μL)	0.088 ± 0.015^↓↓^	0.078 ± 0.012	0.069 ± 0.006	0.074 ± 0.013	0.075 ± 0.021	0.073 ± 0.018	0.038 ± 0.005**	0.040 ± 0.008**	0.019 ± 0.007**
Eosinophils (%)	1.79 ± 0.21^↓↓^	1.78 ± 0.25	1.59 ± 0.16	1.48 ± 0.17	1.48 ± 0.32	1.91 ± 0.55	0.93 ± 0.07*	1.35 ± 0.23*	1.39 ± 0.47
Basophils (K/μL)	0.005 ± 0.002	0.008 ± 0.003	0.006 ± 0.002	0.005 ± 0.002	0.008 ± 0.002	0.004 ± 0.002	0.008 ± 0.002	0.004 ± 0.002	0.000 ± 0.000*
Basophils (%)	0.14 ± 0.02	0.19 ± 0.02	0.15 ± 0.03	0.14 ± 0.03	0.13 ± 0.03	0.13 ± 0.05	0.15 ± 0.03	0.15 ± 0.03	0.05 ± 0.02**
Large Unstained Cells (K/mL)	0.025 ± 0.004^↓^	0.025 ± 0.003	0.025 ± 0.004	0.024 ± 0.003	0.030 ± 0.005	0.021 ± 0.005	0.024 ± 0.004	0.014 ± 0.002	0.016 ± 0.003
Large Unstained Cells (%)	0.51 ± 0.04	0.59 ± 0.07	0.54 ± 0.06	0.46 ± 0.03	0.56 ± 0.06	0.53 ± 0.06	0.58 ± 0.06	0.49 ± 0.05	1.04 ± 0.12**

Data are presented as mean ± standard error of the mean. CPS-Cyclophosphamide. See CEBS, Summary Table M03 for group sizes and CEBS, Summary Table I02 for unscheduled deaths. Decreasing trend with increasing dose of PAC, at ^↓^p < 0.05 or ^↓↓^p < 0.01. Significantly different from the vehicle control group at *p < 0.05 or **p < 0.01.

**TABLE 7:** Immunophenotypes in spleen cells of female B6C3F1/N mice treated orally for 28 days with benzo(a)pyrene.

	Benzo(a)pyrene (mg/kg bodyweight/day)								CPS 50 mg/kg bodyweight/day
	0	2	5	9	19	38	75	150	
Absolute count (mean ± SEM)									
Lymphocytes/10^6^	99.155 ± 4.852^↓↓^	88.101 ± 8.070*	83.807 ± 4.681*	78.559 ± 6.835*	67.691 ± 4.611**	66.955 ± 6.411**	59.255 ± 4.143**	48.740 ± 2.870**	25.570 ± 1.495**
Total T Cells/10^6^	45.082 ± 2.292^↓↓^	41.137 ± 4.346	40.176 ± 2.765	39.234 ± 3.705	30.812 ± 2.321**	27.777 ± 2.593**	26.486 ± 1.650**	22.921 ± 1.565**	15.583 ± 1.143**
CD4^+^ T Cells/10^6^	26.876 ± 1.571^↓↓^	24.716 ± 2.743	24.131 ± 1.666	23.218 ± 2.208	18.176 ± 1.355**	16.940 ± 1.610**	16.227 ± 1.017**	13.448 ± 0.918**	8.123 ± 0.679**
CD8^+^ T cells/10^6^	16.508 ± 0.694^↓↓^	14.729 ± 1.473	14.399 ± 1.029	14.454 ± 1.391	11.369 ± 0.884**	9.836 ± 0.886**	9.391 ± 0.589**	8.815 ± 0.619**	6.408 ± 0.601**
B Cells/10^6^	42.581 ± 2.187^↓↓^	38.035 ± 3.086	35.938 ± 1.857*	32.090 ± 2.599*	31.093 ± 2.390*	34.282 ± 3.529*	29.084 ± 2.631**	23.315 ± 1.317**	6.423 ± 0.282**
NK Cells/10^6^	4.680 ± 0.675^↓↓^	3.696 ± 0.622	2.994 ± 0.273	2.761 ± 0.541*	2.014 ± 0.221**	1.883 ± 0.325**	1.306 ± 0.140**	0.889 ± 0.091**	1.484 ± 0.845**
Mono/Mac/10^6^	1.849 ± 0.098^↓↓^	1.529 ± 0.123	1.381 ± 0.077**	1.254 ± 0.126**	1.107 ± 0.075**	1.012 ± 0.109**	0.769 ± 0.083**	0.441 ± 0.035**	0.327 ± 0.078**
Neutrophils/10^6^	0.469 ± 0.041^↓↓^	0.375 ± 0.032	0.304 ± 0.037*	0.327 ± 0.035*	0.316 ± 0.044**	0.276 ± 0.033**	0.223 ± 0.039**	0.196 ± 0.017**	0.215 ± 0.167*
Eosinophils/10^6^	0.149 ± 0.011^↓↓^	0.140 ± 0.010	0.101 ± 0.005*	0.123 ± 0.014*	0.097 ± 0.015**	0.076 ± 0.011**	0.062 ± 0.013**	0.037 ± 0.006**	0.022 ± 0.008**
T:B Cell Ratio	1.064 ± 0.038^↓↓^	1.087 ± 0.075	1.118 ± 0.050	1.223 ± 0.059	1.009 ± 0.074	0.829 ± 0.049	0.944 ± 0.073	0.983 ± 0.040	2.439 ± 0.163**
CD4:CD8 T Cell Ratio	1.622 ± 0.038	1.677 ± 0.046	1.678 ± 0.031	1.609 ± 0.048	1.603 ± 0.023	1.718 ± 0.053	1.732 ± 0.033	1.531 ± 0.045	1.281 ± 0.050**
Relative percentages (Mean ± SEM)									
Total T Cells (%)	45.529 ± 0.995	46.479 ± 1.823	47.766 ± 1.145	49.873 ± 1.251	45.566 ± 1.597	41.805 ± 1.379	45.091 ± 1.793	46.903 ± 0.952	61.683 ± 3.973*
CD4^+^ T Cells (%)	59.466 ± 0.540	59.955 ± 0.545	60.043 ± 0.436	59.129 ± 0.713	59.011 ± 0.354	60.876 ± 0.678	61.279 ± 0.395	58.693 ± 0.647	51.821 ± 1.415**
CD8^+^ T Cells (%)	36.744 ± 0.506	35.876 ± 0.680	35.829 ± 0.408	36.886 ± 0.648	36.856 ± 0.337	35.571 ± 0.682	35.449 ± 0.451	38.463 ± 0.654	40.768 ± 1.526
B Cells (%)	42.960 ± 0.876^↑↑^	43.525 ± 1.638	43.010 ± 0.892	41.138 ± 1.097	45.943 ± 1.576	50.998 ± 1.437**	48.750 ± 1.856**	47.955 ± 0.913**	25.376 ± 0.890**
NK Cells (%)	4.674 ± 0.613^↓↓^	4.086 ± 0.376	3.596 ± 0.305	3.389 ± 0.499	2.944 ± 0.196*	2.735 ± 0.308**	2.170 ± 0.104**	1.819 ± 0.146**	5.184 ± 2.664
Mono/Mac (%)	1.794 ± 0.032^↓↓^	1.701 ± 0.061	1.619 ± 0.052**	1.549 ± 0.052**	1.618 ± 0.061**	1.481 ± 0.068**	1.276 ± 0.072**	0.893 ± 0.030**	1.198 ± 0.215
Neutrophils (%)	0.458 ± 0.039	0.420 ± 0.028	0.371 ± 0.065	0.412 ± 0.040	0.460 ± 0.051	0.410 ± 0.037	0.364 ± 0.042	0.399 ± 0.022	0.682 ± 0.496*
Eosinophils (%)	0.147 ± 0.012^↓↓^	0.157 ± 0.008	0.120 ± 0.007	0.153 ± 0.012	0.137 ± 0.017	0.110 ± 0.009	0.103 ± 0.017*	0.074 ± 0.008**	0.078 ± 0.022*

Data are presented as mean ± SEM (standard error of the mean); CPS-cyclophosphamide; Mono/Mac-Monocyte/Macrophage. N = 8 per treatment group. Significant increasing (^↑↑^p < 0.01) or decreasing (^↓↓^p < 0.01) trend with increasing dose of B(*a*)P. Significantly different from the vehicle control group at *p < 0.05 or **p < 0.01.

### Antibody responses

**FIGURE 1:**
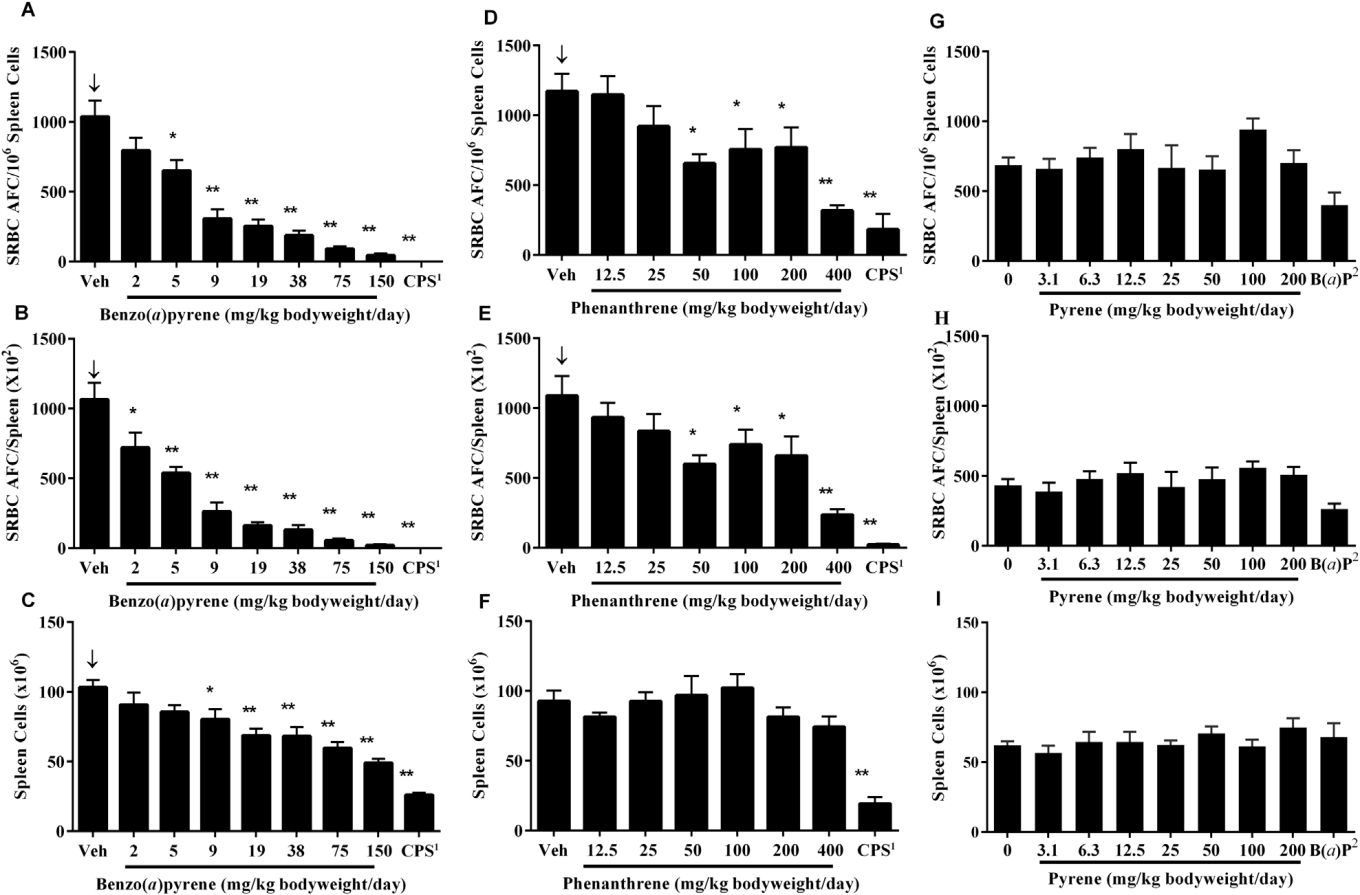
AFC response to SRBC in mice treated with B(*a*)P, phenanthrene or pyrene. Data are shown as AFC/10^6^ spleen cells **(A, D, G)**, AFCs per spleen **(B, E, H)** and total spleen cells **(C, F, I)**. Each value represents the mean ± SEM of 5-8 mice (see CEBS Summary Tables M07 for group size and I02 for unscheduled deaths). ^1^Cyclophosphamide (CPS; positive control) was administered at a dose of 50 mg/kg bodyweight/day in saline, once per day via intraperitoneal (IP) injection on Days 24–27 for the B(*a*)P and phenanthrene studies. ^2^Benzo(*a*)pyrene [B(*a*)P; positive control] was administered on the same schedule as pyrene at a dose of 10 mg/kg bodyweight/day. ^↓^Indicates a significant decreasing trend with increasing dose of PAC (p < 0.01). Significantly different from the vehicle control group at *p < 0.05 or **p < 0.01.

The T-dependent antibody response (TDAR) to SRBC following PAC treatment in mice was assessed 4 days following immunization by enumerating individual antibody forming cells (AFC) in the spleen (CEBS Summary Table M07) as well as determining the titer of IgM antibodies against SRBC in serum (CEBS Summary Table M08). The AFC responses are presented following normalization as per 10^6^ spleen cells and per total spleen cells which allows for discrimination of direct functional effects from the potential impact of changes in cellularity of the spleen on the TDAR. There were significant negative exposure related trends for the AFC response to SRBC following B(*a*)P treatment (Figure 1). In addition, the number of AFC/10^6^ spleen cells and AFC/total spleen cells were significantly decreased in mice treated with ≥5 mg/kg bodyweight/day and ≥2 mg/kg bodyweight/day, respectively (Figures 1A,B). There was also a significant decrease in nucleated spleen cells numbers at doses ≥9 mg/kg bodyweight/day B(*a*)P (Figure 1C). Treatment with phenanthrene also resulted in a significant negative exposure related trend in the SRBC AFC response (Figure 1D) with significant decreases in AFC/10^6^ spleen cells (Figure 1D) and AFC/spleen (Figure 1E) in mice treated with ≥50 mg/kg bodyweight/day phenanthrene relative to the vehicle control group. In contrast to B(*a*)P, treatment with phenanthrene did not affect the total number of nucleated spleen cells (Figure 1F). Pyrene treatment did not affect the SRBC AFC response (Figures 1G–I). The positive control groups performed as expected, with both the number of AFCs and spleen cells markedly reduced following treatment with CPS and 10 mg/kg bodyweight/day B(*a*)P.

**FIGURE 2:**
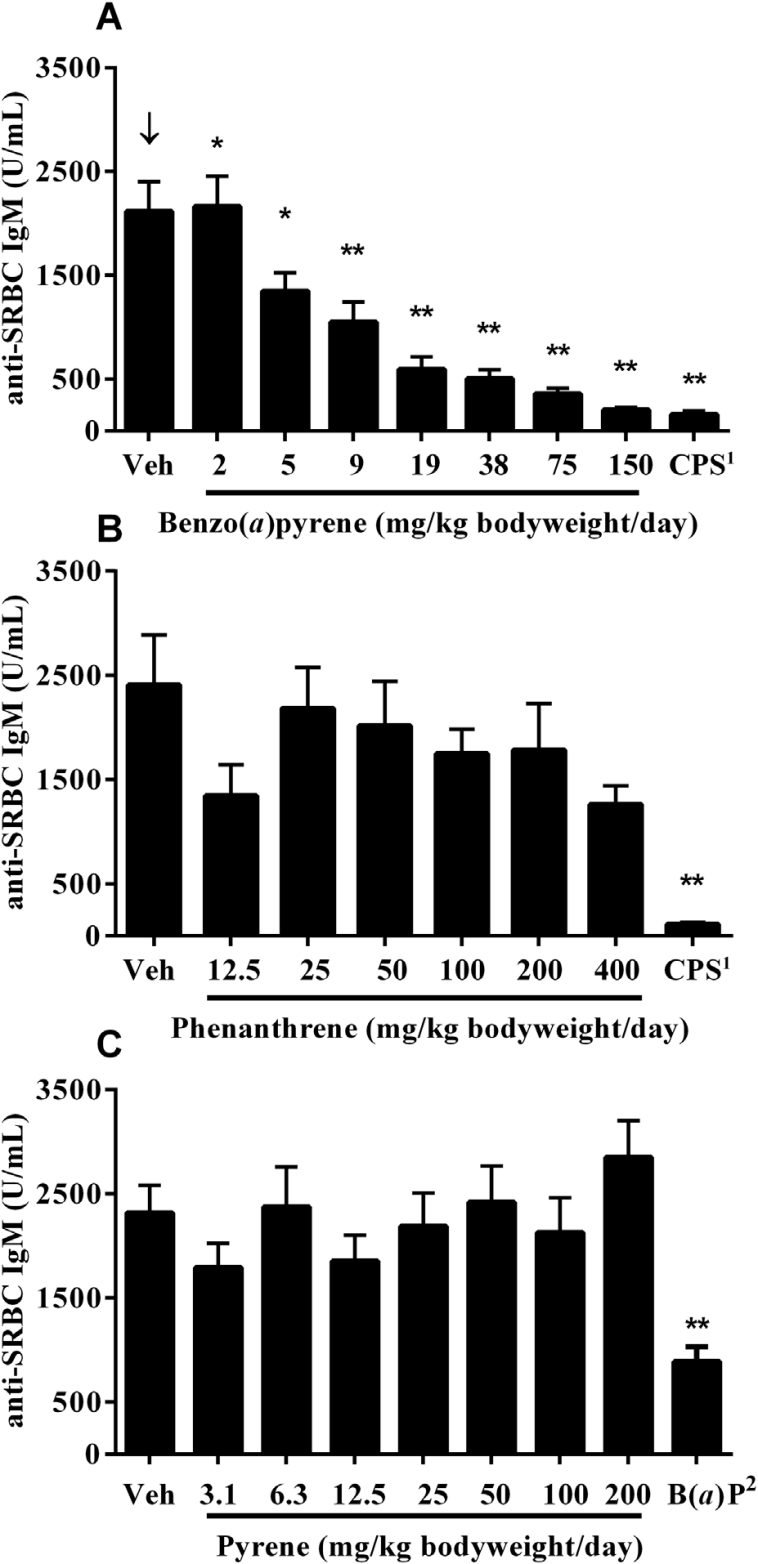
Anti-SRBC IgM antibody titers in serum 4 days following immunization of mice treated orally for 28 days with **(A)** B(*a*)P **(B)** phenanthrene, or **(C)** pyrene. Each value represents the Mean ± SEM of 5-8 mice (see CEBS Summary Tables M08 for group sizes and I02 for unscheduled deaths). ^1^Cyclophosphamide (CPS) was administered at a dose of 50 mg/kg bodyweight/day in saline, once per day via intraperitoneal (IP) injection on Days 24 (day of immunization with SRBC) to 27 for the B(*a*)P and phenanthrene studies. ^2^Benzo(*a*)pyrene [B(*a*)P; positive control] was administered on the same schedule as pyrene at a dose of 10 mg/kg body. ^↓^Indicates a significant decreasing trend with increasing dose of PAC (p < 0.01). Significantly different from the vehicle control group at *p < 0.05 or **p < 0.01.

A significant decrease, with increasing test concentrations of B(*a*)P, was observed in titers of serum anti-SRBC IgM antibodies, with statistically significant decreases observed in mice treated with ≥5 mg/kg bodyweight/day (Figure 2A). Treatment with phenanthrene (Figure 2B) or pyrene (Figure 2C) did not affect anti-SRBC IgM antibody titers. A marked decrease was observed in the positive control groups following treatment with CPS and 10 mg/kg bodyweight/day B(*a*)P.

### Immunophenotyping

Spleen cells were collected and stained with fluorescently labeled antibodies to cell surface markers for immunophenotypic analyses. Cell types enumerated included total lymphocytes, B cells, T cells, CD4^+^ lymphocytes, CD8^+^ lymphocytes, NK cells, monocytes/macrophages, eosinophils and neutrophils. In addition, CD4^+^:CD8^+^ and T:B cell ratios were determined. The results are presented as both absolute numbers and the relative percentage of nucleated spleen cells. Decreases were observed in the absolute number of all immunophenotypes examined following B(*a*)P treatment (Table 7). Total lymphocytes in the spleen were significantly decreased in all B(*a*)P treatment groups relative to the vehicle control group. Statistically significant decreases in the number of T cells, CD4^+^ T cells, and CD8^+^ T cells occurred at doses ≥19 mg/kg bodyweight/day, NK cells at doses ≥9 mg/kg bodyweight/day, and B cells, monocyte/macrophage, neutrophils, and eosinophils at doses ≥5 mg/kg bodyweight/day B(*a*)P. In contrast, there was little, if any, effect of B(*a*)P treatment on CD4^+^:CD8^+^ or T:B cell ratios compared to the vehicle control group suggesting a lack of selectivity for these immune cell populations. There was little change when analyzed as relative percentages with only small decreases in the percentage of NK cells (≥19 mg/kg bodyweight/day), monocyte/macrophage (≥5 mg/kg bodyweight/day), and eosinophils (≥75 mg/kg bodyweight/day) while the percentage of B cells showed a small but significant increase at dose levels ≥38 mg/kg bodyweight/day B(*a*)P.

There were minor changes in immune cell populations in the spleen following oral treatment of mice with phenanthrene (Table 8) and pyrene (Table 9) This included a significant decrease in eosinophils in the 400 mg/kg bodyweight/day phenanthrene treatment group and a slight, but significant, decrease in the T:B cell ratio in splenocytes of mice treated with ≥25 mg/kg bodyweight/day pyrene. The shift in T:B cell ratio in mice treated with pyrene corresponded with an increasing trend observed for the B cell population (absolute and relative) and a decreasing trend for T cells (relative) with the relative counts being significant for both populations in mice treated with ≥25 mg/kg bodyweight/day pyrene. Treatment of mice with ≥50 mg/kg bodyweight/day pyrene also decreased the relative percentage of eosinophils. These changes caused by phenanthrene and pyrene were small and unlikely to have a biological impact.

**TABLE 8:** Immunophenotypes in spleen cells of female B6C3F1/N mice treated orally for 28 days with phenanthrene.

	Phenanthrene (mg/kg bodyweight/day)								CPS 50 mg/kg bodyweight/day
	0	12.5	25	50	100	200	400	800^a^	
Absolute count (mean ± SEM)									
Lymphocytes/10^6^	89.390 ± 6.952	78.354 ± 3.342	89.042 ± 5.948	94.686 ± 13.193	98.963 ± 9.229	78.358 ± 6.561	71.611 ± 6.841	N/A	18.706 ± 4.595**
Total T Cells/10^6^	43.648 ± 2.634	39.986 ± 2.417	44.806 ± 2.821	48.806 ± 7.551	50.637 ± 5.769	40.073 ± 4.667	35.494 ± 4.511	N/A	12.973 ± 3.187**
CD4^+^ T Cells/10^6^	25.692 ± 1.520	23.919 ± 1.519	26.832 ± 2.274	29.967 ± 5.104	30.538 ± 3.769	23.302 ± 2.734	20.397 ± 2.805	N/A	6.730 ± 1.700**
CD8^+^ T cells/10^6^	15.265 ± 0.870	13.819 ± 0.790	15.403 ± 0.502	16.214 ± 2.110	17.290 ± 1.755	14.607 ± 1.797	13.022 ± 1.536	N/A	5.299 ± 1.354**
B Cells/10^6^	38.627 ± 3.955	31.656 ± 1.235	37.180 ± 2.903	38.454 ± 5.001	40.356 ± 2.969	31.637 ± 2.986	29.559 ± 2.646	N/A	3.697 ± 1.155**
NK Cells/10^6^	3.106 ± 0.235	3.142 ± 0.182	3.316 ± 0.287	3.202 ± 0.391	3.566 ± 0.472	2.942 ± 0.286	2.824 ± 0.359	N/A	0.838 ± 0.284**
Mono/Mac/10^6^	1.471 ± 0.125	1.334 ± 0.056	1.443 ± 0.161	1.602 ± 0.226	1.595 ± 0.190	1.188 ± 0.100	1.110 ± 0.116	N/A	0.172 ± 0.038**
Neutrophils/10^6^	0.449 ± 0.066	0.317 ± 0.035	0.324 ± 0.039	0.430 ± 0.070	0.425 ± 0.052	0.387 ± 0.065	0.313 ± 0.052	N/A	0.091 ± 0.042**
Eosinophils/10^6^	0.109 ± 0.009	0.085 ± 0.011	0.085 ± 0.014	0.104 ± 0.021	0.104 ± 0.013	0.097 ± 0.015	0.067 ± 0.010*	N/A	0.028 ± 0.015**
T:B Cell Ratio	1.164 ± 0.061	1.267 ± 0.069	1.213 ± 0.042	1.250 ± 0.077	1.244 ± 0.072	1.300 ± 0.137	1.206 ± 0.137	N/A	3.994 ± 0.625**
CD4:CD8 T Cell Ratio	1.683 ± 0.027	1.730 ± 0.026	1.739 ± 0.117	1.786 ± 0.074	1.754 ± 0.050	1.606 ± 0.054	1.550 ± 0.059	N/A	1.267 ± 0.088*
Relative Percentages (Mean ± SEM)									
Total T Cells (%)	49.273 ± 1.190	50.898 ± 1.273	50.396 ± 0.721	50.801 ± 1.392	50.792 ± 1.370	50.857 ± 3.038	48.754 ± 2.417	N/A	67.740 ± 3.511**
CD4^+^ T Cells (%)	58.888 ± 0.388	59.777 ± 0.306	59.608 ± 1.439	60.425 ± 0.952	60.096 ± 0.704	58.153 ± 0.820	57.019 ± 0.972	N/A	50.126 ± 1.898**
CD8^+^ T Cells (%)	35.028 ± 0.394^↑^	34.592 ± 0.388	34.690 ± 1.569	34.078 ± 0.824	34.320 ± 0.549	36.353 ± 0.806	36.981 ± 0.839	N/A	40.246 ± 1.957
B Cells (%)	42.764 ± 1.170	40.508 ± 1.247	41.638 ± 0.776	41.204 ± 1.318	41.132 ± 1.291	40.607 ± 2.825	42.076 ± 2.423	N/A	19.470 ± 2.335**
NK Cells (%)	3.481 ± 0.112	4.013 ± 0.184	3.712 ± 0.158	3.451 ± 0.134	3.566 ± 0.166	3.757 ± 0.187	3.927 ± 0.301	N/A	4.451 ± 1.046
Mono/Mac (%)	1.581 ± 0.014	1.636 ± 0.026	1.541 ± 0.085	1.657 ± 0.046	1.548 ± 0.053	1.468 ± 0.066	1.493 ± 0.043	N/A	1.000 ± 0.195*
Neutrophils (%)	0.514 ± 0.111	0.387 ± 0.035	0.350 ± 0.039	0.444 ± 0.028	0.424 ± 0.055	0.476 ± 0.067	0.414 ± 0.049	N/A	0.833 ± 0.558
Eosinophils (%)	0.120 ± 0.010	0.104 ± 0.014	0.097 ± 0.018	0.106 ± 0.009	0.103 ± 0.011	0.119 ± 0.016	0.089 ± 0.010	N/A	0.270 ± 0.184

Data are presented as mean ± SEM (standard error of the mean); CPS-cyclophosphamide; Mono/Mac-Monocyte/Macrophage.

^a^ Group removed due to overt toxicity. N = 5-8 per treatment group (see CEBS, Summary Table M06 for group sizes and CEBS, Summary Table I02 for unscheduled deaths). Significant increasing (^↑^p < 0.05) trend with increasing dose of phenanthrene. Significantly different from the vehicle control group at *p < 0.05 or **p < 0.01.

**TABLE 9:** Immunophenotypes in spleen cells of mice treated orally for 28 days with pyrene.

	Pyrene (mg/kg bodyweight/day)								B(*a*)P 10 mg/kg bodyweight/day
	0	3.1	6.3	12.5	25	50	100	200	
Absolute Count (Mean ± SEM)									
Lymphocytes/10^6^	60.240 ± 2.818	53.500 ± 5.470	61.591 ± 4.541	62.384 ± 6.783	60.716 ± 3.446	68.572 ± 4.908	59.845 ± 4.724	68.760 ± 5.342	58.416 ± 8.204
Total T Cells/10^6^	27.021 ± 1.282	22.888 ± 2.740	26.224 ± 2.274	26.339 ± 2.578	25.070 ± 1.401	28.720 ± 1.932	25.229 ± 2.047	28.050 ± 2.206	24.696 ± 3.452
CD4^+^ T Cells/10^6^	16.079 ± 0.974	13.801 ± 1.800	15.911 ± 1.412	15.848 ± 1.552	15.233 ± 0.903	17.564 ± 1.238	15.147 ± 1.222	16.771 ± 1.271	15.083 ± 2.248
CD8^+^ T cells/10^6^	9.469 ± 0.473	8.021 ± 0.822	9.086 ± 0.772	9.203 ± 0.881	8.627 ± 0.457	9.797 ± 0.602	8.893 ± 0.727	9.880 ± 0.808	8.534 ± 1.072
B Cells/10^6^	28.702 ± 1.466^↑^	25.419 ± 2.950	30.840 ± 2.015	31.482 ± 3.840	31.189 ± 1.865	34.821 ± 2.813	30.497 ± 2.411	35.850 ± 2.932	29.836 ± 4.268
NK Cells/10^6^	1.089 ± 0.055	0.986 ± 0.122	1.141 ± 0.055	1.226 ± 0.133	1.191 ± 0.073	1.268 ± 0.104	1.061 ± 0.095	1.196 ± 0.106	0.982 ± 0.141
Mono/Mac/10^6^	1.518 ± 0.074	1.415 ± 0.154	1.552 ± 0.123	1.575 ± 0.162	1.558 ± 0.119	1.744 ± 0.131	1.441 ± 0.101	1.702 ± 0.132	1.330 ± 0.186
Neutrophils/10^6^	0.091 ± 0.009	0.082 ± 0.019	0.084 ± 0.008	0.121 ± 0.030	0.119 ± 0.011	0.120 ± 0.011	0.078 ± 0.009	0.088 ± 0.011	0.106 ± 0.024
Eosinophils/10^6^	0.224 ± 0.010	0.229 ± 0.022	0.226 ± 0.014	0.238 ± 0.029	0.222 ± 0.029	0.212 ± 0.016	0.171 ± 0.012	0.232 ± 0.017	0.188 ± 0.029
T:B Cell Ratio	0.945 ± 0.024^↓↓^	0.899 ± 0.030	0.846 ± 0.031	0.866 ± 0.052	0.806 ± 0.019*	0.836 ± 0.035*	0.830 ± 0.023*	0.787 ± 0.023**	0.835 ± 0.022**
CD4:CD8 T Cell Ratio	1.695 ± 0.035	1.694 ± 0.054	1.747 ± 0.032	1.719 ± 0.033	1.764 ± 0.032	1.787 ± 0.026	1.707 ± 0.029	1.703 ± 0.015	1.730 ± 0.057
Relative Percentages (Mean ± SEM)									
Total T Cells (%)	44.873 ± 0.576^↓↓^	43.840 ± 0.806	42.356 ± 0.792	42.746 ± 1.311	41.317 ± 0.518*	42.044 ± 0.937*	42.183 ± 0.677*	40.841 ± 0.712**	42.395 ± 0.590*
CD4^+^ T Cells (%)	59.851 ± 0.495	59.874 ± 0.701	60.573 ± 0.423	60.1340 ± 0.434	60.696 ± 0.417	61.070 ± 0.339	60.076 ± 0.417	59.876 ± 0.246	60.504 ± 0.734
CD8^+^ T Cells (%)	35.377 ± 0.469	35.469 ± 0.691	34.726 ± 0.396	35.053 ± 0.469	34.453 ± 0.412	34.213 ± 0.324	35.245 ± 0.394	35.175 ± 0.206	35.135 ± 0.717
B Cells (%)	47.601 ± 0.595^↑↑^	48.926 ± 0.739	50.315 ± 0.877	49.949 ± 1.341	51.343 ± 0.590*	50.583 ± 0.924*	50.953 ± 0.640**	52.044 ± 0.673**	50.899 ± 0.624**
NK Cells (%)	1.826 ± 0.107	1.883 ± 0.026	1.893 ± 0.099	1.966 ± 0.064	1.961 ± 0.046	1.859 ± 0.100	1.765 ± 0.048	1.738 ± 0.063	1.674 ± 0.045
Mono/Mac (%)	2.449 ± 0.038^↓^	2.552 ± 0.033	2.404 ± 0.080	2.446 ± 0.036	2.480 ± 0.077	2.475 ± 0.045	2.367 ± 0.044	2.291 ± 0.052	1.975 ± 0.034**
Neutrophils (%)	0.145 ± 0.008	0.141 ± 0.017	0.129 ± 0.006	0.175 ± 0.022	0.190 ± 0.012	0.171 ± 0.012	0.126 ± 0.010	0.117 ± 0.010	0.148 ± 0.023
Eosinophils (%)	0.363 ± 0.013^↓↓^	0.422 ± 0.020	0.356 ± 0.019	0.363 ± 0.021	0.352 ± 0.032	0.301 ± 0.007*	0.282 ± 0.009*	0.316 ± 0.017*	0.282 ± 0.019*

Data are presented as mean ± SEM (standard error of the mean); B(*a*)P-benzo(*a*)pyrene; Mono/Mac-Monocyte/Macrophage. N = 7-8 per treatment group (see CEBS, Summary Table M06 for group sizes and CEBS, Summary Table I02 for unscheduled deaths). Significant increasing (^↑^p < 0.05, ^↓^p < 0.05, ^↓↓^p < 0.01) trend with increasing dose of pyrene. Significantly different from the vehicle control group at *p < 0.05 or **p < 0.01.

All immune cell populations in the spleen were significantly reduced in mice treated with the positive control CPS, which is consistent with the splenic atrophy and reduced splenocyte numbers.

### Bone marrow

There were no effects following B(*a*)P, phenanthrene, or pyrene treatment on total cellularity (CEBS Summary Table M17) or histopathology of the bone marrow (CEBS Summary Tables PA10, PA14, and PA18), relative to vehicle controls.

### Sensitivity of the TDAR and selected immunophenotypes to PAC treatment

The quantitative relationship between the TDAR, selected immunophenotypes, and selected organ weights to PAC treatment were determined by benchmark dose (BMD) modeling using 1SD of the control mean as the reference value (Table 10). All endpoints provided BMD estimates for B(*a*)P whereas fewer endpoints showed enough change to successfully model using 1SD of the control for phenanthrene and pyrene treatments. B(*a*)P was the most potent PAC examined (BMDs ranged from 2.00 to 144.48 mg/kg bodyweight/day) as all BMDs were lower (all but liver weight were >1 order of magnitude lower) than the corresponding BMDs for phenanthrene (BMDs ranged from 29.30 to 1044.41 mg/kg bodyweight/day) and pyrene (207.27–1251.45 mg/kg bodyweight/day). The TDAR to SRBC was the most sensitive parameter to B(*a*)P treatment with the BMD being 3.22 mg/kg bodyweight/day and 2.00 mg/kg bodyweight/day for the AFC/10^6^ spleen cells and AFC/spleen, respectively. Serum levels of anti-SRBC IgM was approximately 2 times less sensitive to B(*a*)P with a BMD of 7.33 mg/kg bodyweight/day. The impact of B(*a*)P on T-cell, B-cell, macrophage/monocyte, and NK cell populations in the spleen were also less than the AFC response with BMDs ranging from 6.79–22.07 mg/kg bodyweight/day. The least sensitive parameters examined were organ weights. Spleen weight had a BMD of 74.47 mg/kg bodyweight/day which was less sensitive than the impact on immune cell populations from the spleen. Likewise, the organ weight BMDs of 28.19 mg/kg bodyweight/day for the thymus and 144.48 mg/kg bodyweight/day for the liver were substantially higher than for the TDAR in animals treated with B(*a*)P.

**TABLE 10:** Benchmark dose modeling for organ weights, cell population numbers, and antibody responses in mice treated orally with benzo(*a*)pyrene, phenanthrene, or pyrene.

Endpoint	BMD	BMD_L_	Model
Benzo(*a*)pyrene			
Liver weight	144.48	66.76	Exponential M3
Thymus weight	28.19	21.38	Exponential M3
Spleen Weight	74.47	52.36	Exponential M3
AFC/10^6^ cells	3.22	2.02	Hill
AFC/spleen	2.00	1.41	Hill
anti-SRBC IgM	7.33	4.36	Hill
Total Lymphocytes	6.85	2.75	Hill
Total T-cells	9.93	5.23	Exponential M5
CD4^+^ T-cells	16.94	6.84	Hill
CD8^+^ T-cells	9.36	5.11	Exponential M5
B-cells	7.52	1.81	Hill
NK Cells	22.07	9.02	Hill
Mono/Mac Cells	6.79	3.39	Polynomial 3
Phenanthrene			
Liver weight	No Viable Model		
Thymus weight	1044.41	377.58	Linear
Spleen Weight	501.01	379.40	Polynomial 3
AFC/10^6^ cells	No Viable Model		
AFC/spleen	29.30	6.36	Power
anti-SRBC IgM	502.95	263.08	Power
Total Lymphocytes	No Viable Model		
Total T-cells	No Viable Model		
CD4^+^ T-cells	No Viable Model		
CD8^+^ T-cells	No Viable Model		
B-cells	No Viable Model		
NK Cells	797.51	328.40	Linear
Mono/Mac Cells	No Viable Model		
Pyrene			
Liver weight	255.47	167.49	Polynomial 3
Thymus weight	No Viable Model		
Spleen Weight	1251.45	251.52	Linear
AFC/10^6^ cells	855.90	208.64	Linear
AFC/spleen	416.66	178.75	Linear
anti-SRBC IgM	255.13	141.62	Linear
Total Lymphocytes	No Viable Model		
Total T-cells	555.09	199.98	Linear
CD4^+^ T-cells	624.09	207.68	Linear
CD8^+^ T-cells	447.46	183.29	Linear
B-cells	233.89	132.86	Linear
NK Cells	216.96	201.95	Exponential M3
Mono/Mac Cells	207.27	198.01	Exponential M3

AFC, antibody forming cell; NK, Natural Killer, Mono/Mac–monocyte/macrophage; NC-noncalculable.

^a^ Data presented as mg/kg bodyweight/day of the respective PAC. BMDS, Online (US EPA, 2024) was used to model the dose-response relationships using a reference value of 1 standard deviation of the control mean. Data are presented as the dose (mg/kg bodyweight/day) of the respective PAC, corresponding to the reference value of the response.

## Discussion

The immune response profile was characterized for one potent PAC, B(*a*)P, and two PACs at the other end of the potency spectrum, phenanthrene and pyrene. These studies further support that B(*a*)P is a potent immunotoxic chemical with apparent selectivity towards disruption of humoral immunity. Several new insights into the mode of action of B(*a*)P on the immune system can be drawn from these data. Despite atrophy and cell loss of central and peripheral lymphoid organs (i.e., spleen, thymus, and mesenteric lymph node) following B(*a*)P treatment, there were no major effects identified on peripheral blood cells, as assessed by WBC counts and differentials. This would imply that B(*a*)P may interfere with late-stage lymphoid cell maturation that occurs predominantly in peripheral lymphoid organs. While the bone marrow is responsible for producing pro-lymphocytes, subsequently these cells migrate to peripheral lymphoid organs where they differentiate into immunocompetent B cells or T cells in an antigen-independent manner or the lymph node where they encounter antigen (Goldsby et al., 2003; Kats, 1977). Thus, while immune cells are generated, it is possible that B(*a*)P interferes with either cell migration to or colonization of secondary lymphoid organs. Holladay and Smith (1995), conducting studies in which differentiation markers for pro-B and pro-T cells were examined following B(*a*)P treatment, also concluded that late-stage maturation is compromised, although they noted that the effect may also be at the level of the bone marrow stem cell as well as central lymphoid organs as there was some depletion of bone marrow prolymphocytic cells. The present study showed that B(*a*)P treatment did not affect total cellularity or histopathology of the bone marrow, but these are relatively crude endpoints.

Immunophenotyping data showed that a significant loss in all immune cell types occurred in the spleen, including lymphocytes, NK cells, monocytes/macrophages and neutrophils following B(*a*)P treatment. This non-selective leukopenia was most evident when comparing absolute values to relative percentages where, in contrast to absolute values, there was no major effect on relative percentages in any cell population. It is important to note, however, that spleen cell depletion is likely not solely responsible for suppression of the TDAR as low dose levels of B(*a*)P, where spleen cell depletion was not present, still showed significant antibody suppression. This also occurred in phenanthrene studies where antibody responses were decreased without evidence of any cell depletion in central lymphoid organs.

The primary TDAR is a complex immune response that involves antigen processing and presentation by antigen presenting cells including dendritic cells and macrophages leading to training of CD4^+^ T cells and B cells to recognize the antigen, in this case SRBC. A benchmark dose modeling approach was used to generate BMDs for the immune endpoints investigated following PAC treatment. The BMD model is a statistical approach used to determine the concentration that causes a predefined effect size in the parameters being studied and can be used to rank sensitivities. The BMD approach is preferred by many regulatory agencies, including the US EPA (US EPA, 2024) for generating points of departure for human health risk assessment and establishment of regulatory action limits. Analyses of sensitivities of the immune parameters examined in this study using BMS showed that the TDAR was the most sensitive endpoint found for both B(*a*)P and phenanthrene. Regarding cell numbers, macrophages and B-cells showed the same order of magnitude sensitivity to B(*a*)P treatment. Interestingly, CD4^+^ T-helper cell numbers were about 2 times less sensitive to B(*a*)P treatment than macrophages and B cells. These data suggest that macrophages and B cells may be more sensitive to the effects of B(*a*)P than CD4^+^ T-helper cells and the suppression of the TDAR may be driven predominantly through impacts on macrophages and B cells. Acute lymphopenia is known to result in marked lymphoproliferation and is considered a homeostatic response to the depletion of lymphocytes (Takada and Jameson, 2009). This compensatory proliferation drives activation of naïve T cells and favors expansion of regulatory T cells, an outcome that may limit downstream immune responses (Eldershaw et al., 2021). Therefore, it is possible that immune cell depletion observed following B(*a*)P treatment may trigger lymphoproliferation in favor of suppressive regulatory T cells, thereby contributing to the deficit in humoral immunity.

Several studies have suggested an association exists between immunotoxicity and carcinogenicity stemming from the concept that immune surveillance mechanisms may be altered by immunotoxic substances (Hanahan and Weinberg, 2011; Luster et al., 1992b; Luster et al., 1992a; Smith et al., 2016; Stjernswärd, 1966). This was highlighted in a detailed examination showing a strong correlation (R^2^ = 0.8976) between the relative cancer potency and relative immunotoxicity potency for 9 PACs (Zaccaria and McClure, 2013). Based on data from the later study, the authors suggested that evidence of immunotoxicity could be used to strengthen cancer risk assessments for PACs. While we found that the potent carcinogen, B(*a*)P, was also a potent immunotoxic agent, phenanthrene also displayed immunotoxicity despite having mostly negative carcinogenic data (IARC, 2010). Pyrene did not suppress immune activity and had mostly negative cancer data. However, it should be noted that deficiencies in the cancer database for both phenanthrene and pyrene led to an IARC conclusion of *not classifiable as a human carcinogen* (category 3) (IARC, 2010). Previous evaluation of the immunotoxic potential of PACs found that phenanthrene elicited no immunosuppression and pyrene suppressed the immune response by 30% following a single oral dose of 100 mg/kg in C57BL/6 mice (Silkworth et al., 1995). Ongoing immunotoxicology studies from our laboratories, using 13 individual PACs, should provide additional data to help clarify the correlation between carcinogenic and immunotoxic potencies (manuscript in preparation).

In 2017, the U.S. EPA’s Integrated Risk Information System (IRIS) program released a document that reviewed potential adverse health effects of B(*a*)P (US EPA, 2017). In selecting a proposed overall reference dose, multiple organ/system-specific reference doses were derived for effects identified as potential hazards from B(*a*)P including developmental toxicity, reproductive toxicity, and immunotoxicity. Developmental and reproductive effects were identified as more sensitive to B(*a*)P than the available immunotoxicity outcomes with developmental neurobehavioral effects occurring at doses as low as 0.02 mg/kg (US EPA, 2017). Although all publicly available B(*a*)P immunotoxicity data were evaluated, only thymus weights (Kroese et al., 2001) and baseline serum IgM levels in rats (De Jong et al., 1999), which are not sensitive targets for PAC immunotoxicity, were considered adequate for conducting quantitative risk assessment under currently used benchmark modeling as other endpoints had limitations in their experimental design. As a result, the confidence for the reference dose derived from the immunotoxicity data was low (US EPA, 2017). We believe the dose response curves obtained in the current studies for both B(*a*)P and phenanthrene should readily lend themselves to currently used models (Filipsson et al., 2003) to assess risk for non-cancer endpoints. The present study demonstrated significant suppression of a critical immune function, the TDAR, at the lowest dose tested of 2 mg/kg bodyweight/day B(*a*)P. At this dose level, the TDAR was suppressed by 32% and it is possible that further reduction in the dose would still result in significant immunosuppression bringing the sensitivity of the immune system in line with developmental and reproductive outcomes.

In summary, B(*a*)P produced significant immunotoxicity, in the absence of overt toxicity, as evidenced by dose related decreases in central and peripheral lymphoid organ weights, humoral antibody responses and spleen cell immunophenotypes. These effects occurred in the absence of any apparent effects on bone marrow or peripheral blood cells numbers. Suggestive evidence is also provided that antibody suppression was due partly to a loss in spleen cell numbers, particularly T cells and macrophages, although functional deficits in surviving cells are also likely involved. The most profound effect was noted in the TDAR which was decreased by >75% compared to vehicle controls at a dose of 9 mg/kg bodyweight/day B(*a*)P. Phenanthrene, a putative non-carcinogenic PAH, also inhibited humoral immunity, but was considerably less potent, while pyrene showed no evidence of immunotoxicity. Based on the most sensitive endpoint of AFC/spleen, the BMD of 2.00 mg/kg bodyweight/day B(*a*)P for the AFC/spleen was at the lowest level tested (2 mg/kg bodyweight/day), while the BMD for AFC/spleen for phenanthrene and pyrene were 29.30 and 416.66 mg/kg bodyweight/day, respectively. These studies establish the range of PAC immunotoxicity from severe immunosuppression induced by B(*a*)P to the lack of immunotoxicity resulting from pyrene. These PACs will be important for ranking the immunotoxic potency of additional PACs which will contribute to opportunities for modeling the impact of mixtures on the immune system and correlating immunotoxic with carcinogenic potency.

## Data Availability

The datasets presented in this study can be found in an online repository owned by NIEHS at the following website; https://doi.org/10.22427/NTP-DATA-500-005-004-000-4.
